# SENSEYE: a resource-aware visionary framework for assisting individuals with visual disabilities

**DOI:** 10.1038/s41598-026-51257-9

**Published:** 2026-05-11

**Authors:** Akash Shingha Bappy, Tapio Seppänen, Md Ziaul Hoque

**Affiliations:** 1 Center for Machine Vision and Signal Analysis, University of Oulu, Oulu, Finland; 2 Translational Immunology Research Program, University of Helsinki, Helsinki, Finland

**Keywords:** Engineering, Mathematics and computing

## Abstract

Despite significant recent advances, visual aid systems are still limited by the use of conventional computer vision algorithms, constrained sensor capabilities, high power consumption, and reliance on cloud-based processing, which introduce latency and privacy risks. Current assistive technologies for visually impaired individuals often suffer from a lack of secure and competent communication and an inability to deal with complex computer vision tasks. This paper introduces SENSEYE, a resource-aware visionary framework that employs edge computing with a secure and competent communication mechanism. The proposed architecture integrates IoT Edge and virtual decentralized services in a portable system that is small, cost-effective, and power-efficient. The integration of open AI models with advanced functionality in this system design helps to recognize objects, locate moving obstacles, detect sudden changes, perceive a summary from a live feed, and represent them as audio in real time. SENSEYE integrates real-time object detection, scene comprehension, and global positioning system (GPS)-based navigation into a portable, low-latency device. This system leverages optimized lightweight AI models, e.g., SSD-MobileNetV2 and VILA1.5-3b, to provide accurate environmental awareness and seamless auditory feedback through efficient speech processing. The system also enables secure remote assistance via video streaming and real-time GPS location sharing, ensuring enhanced user safety and connectivity. The evaluations confirm superior accuracy, power efficiency, and responsiveness compared to traditional sensor-based or cloud-reliant systems. Although challenges remain, particularly in constrained sensor capabilities, power efficiency trade-offs, and potential sensory overload, future work will focus on improving wearability, optimizing energy consumption, and advancing multimodal, user-centric AI integration for enhanced accessibility. This paper presents a novel resource-aware edge AI architecture with integrated real-time perception and secure IoT communication, establishing a scalable, privacy-preserving, and interpretable foundation for next-generation assistive systems for visually impaired individuals.

## Introduction

Visual impairment affects hundreds of millions of people worldwide, creating a strong demand for assistive technologies that can enhance independence and safety. Historically, a variety of solutions have been explored, from the classic white cane (introduced in the early 20th century) and guide dogs to tactile systems like Braille for reading. However, they have notable limitations as they require extensive training, have a limited range of detection, and convey only minimal environmental information to the user. Early electronic travel aids (ETAs) attempted to augment mobility by using sensors (e.g., ultrasonic or infrared rangefinders) to detect obstacles and then alert the user via simple feedback such as beeps or vibrations. For example, a “smart” cane with ultrasonic sensors can detect obstacles within a few meters (often around 2m range) and warn the user with sound^1^. However, such sensor-based solutions cannot identify what the obstacle is, may struggle under certain conditions, i.e., infrared sensors can be disrupted by sunlight, and generally provide a one-dimensional awareness (distance to objects) rather than a rich understanding of the scene^2^.

The last decade saw computer vision and artificial intelligence progress enough to create new avenues for assistive technology. Camera systems started appearing that were able to identify text, objects, or faces and report that information to visually impaired users in an auditory fashion. Initial products were things such as Optical Character Recognition (OCR) reading devices and color detectors, but current systems make greater use of deep learning to provide much higher accuracy and flexibility. For example, Microsoft’s Seeing AI mobile app uses computer vision and AI to narrate the world in real time, reading text, and identifying objects or people using a smartphone^3^. Another breakthrough is the OrCam MyEye wearable camera, which clips onto glasses and uses AI to convert visual information (e.g., printed text, faces, or products) into speech on the device, without requiring internet connectivity^4^. These AI-based aids have the potential to provide more descriptive information than sensor-based aids, but usually need a lot of processing power or cloud services. Utilization of cloud connectivity can bring latency, security/privacy problems, and decrease the reliability of the system in case of loss of internet connectivity. Internet of Things (IoT) and edge computing have emerged as pioneers to bridge this gap in assistive technology in recent days. By connecting smart sensors and devices in an IoT framework, one can combine multiple data sources (e.g., camera, GPS, inertial sensors, etc.) to gain a comprehensive view of the user’s context. Edge Computing refers to performing computation near the data source (on local devices or nearby servers) rather than in distant cloud servers. This approach offers several advantages for visual assistance: it reduces response latency and bandwidth usage by processing data on-device, and it keeps sensitive video data local, which enhances privacy^5^. The latest generation of edge hardware (e.g., NVIDIA Jetson, Google Coral, Apple Neural Engine) has the potential to run advanced deep learning models in real time with relatively low power consumption. This means that it is possible to perform tasks, e.g., object detection, scene comprehension, or OCR on a wearable device or handheld unit without the need for continuous cloud connectivity. IoT connectivity basically provides the sensing and communication fabric, and edge computing provides the intelligence close to the user, collectively enabling a new generation of assistive devices that are responsive and smart.

In this study, we propose a resource-aware visionary framework to assist visually impaired users by employing IoT edge computing to overcome the limitations of existing approaches. The main goal is to develop an accessible system capable of interpreting and describing the user environment in real time, thereby facilitating safe navigation and greater independence in daily tasks. Unlike conventional aids, the framework integrates multiple functions, including real-time object recognition and localization, detection of moving obstacles and sudden environmental changes, scene summarization from a live camera feed, and instantaneous auditory feedback, while also providing communication features such as audio or video calls with remote assistants and live GPS location sharing via WiFi or cellular networks, thereby adding a critical safety net for situations beyond automated recognition. In summary, this work advances a resource-aware, edge-first assistive framework that unifies real-time object detection, scene understanding, audio interaction, and connected safety services within a single deployable system. Its primary contribution lies in the system-level co-design and deployment on constrained hardware, supported by comparative analyses of latency, efficiency, and functionality rather than a novel standalone learning algorithm; we explicitly recognize that motion-aware navigation robustness (heading estimation, drift accumulation, and device-pose variability) is not fully resolved in this paper and is therefore treated as a limitation and target for future validation.

The rest of the paper is organized as follows: Section Related works discusses background and related works. Section Methodology describes the proposed framework, including different components. Section Experimental results presents the experimental results and demonstrates the state-of-the-art results. Section Discussion and Section Limitations and future work summarize the main findings relative to prior publications, limitations of the proposed work, and future directions. Finally, conclusive statements are provided in Section Conclusion.

## Related works

Research on assistive technology for visually impaired individuals spans several decades and can be broadly divided into two categories: non-AI-based (sensor-based) solutions and AI-based (computer vision) solutions. In addition, a number of commercial products and services have emerged in recent years, incorporating advancements from both categories. This section summarizes representative prior works in each category, highlighting their approaches and limitations, and discusses how deep learning and edge computing are increasingly shaping modern solutions^6^.

### Non-AI-based solutions

Early technological aids for the blind relied on sensor circuitry and simple logic rather than machine learning or computer vision. The goal of these systems was often to mimic or augment the traditional white cane by detecting obstacles and hazards beyond the cane’s physical reach. A classic example is the ultrasonic sensor-based “smart cane”. In such a system, one or more ultrasonic transducers emit sound waves and measure reflections to estimate the distance to obstacles. When an object is detected within a certain range, the device alerts the user via vibrations, buzzing sounds, or spoken warnings using a text-to-speech module^7^. Numerous research prototypes of ultrasonic canes have been proposed. Dambhare et al. (2011) developed “Smart Stick for the Blind” that combined ultrasonic obstacle detection with GPS for location assistance^8^. Olakanmi et al. (2014) presented an ultrasonic sensor network embedded in a cane, providing voice guidance to indicate an obstacle’s direction and distance^9^. Furthermore, Gbenga et al. (2017) designed a low-cost Arduino-based smart cane with multiple ultrasonic sensors for frontal obstacle and puddle detection; their cane could detect obstacles 2 meters ahead and would beep to alert the user^1^. Elmannai and Elleithy et al. used a tongue electro-tactile device (TED) to provide haptic feedback to the user for transmitting information and navigation^2^.

Although popular, non-AI solutions have broad limitations. Due to a lack of complex image processing, they are incapable of identifying the type of an object or being able to generate semantic information, e.g., the systems know there is something in front of them, but whether it is car, signpost, or human, they cannot state. This context lack can leave the user unconvinced or force them into trial-and-error. Many of the sensors are subjected to face environmental limitations. Infrared sensors are susceptible to being misled by sunlight or reflective surfaces, resulting in false positives or false negatives. Ultrasonic sensors have a limited detection cone and can fail to detect drop-offs or glass barriers consistently, and overestimate distance on sloping or smooth surfaces^10^. Other sensor-based approaches, e.g., RFID tag systems, require infrastructure (tags in the environment) and only work for tagged locations, limiting their range^11^. There are also wearable designs, e.g., sensor belts or vests that give vibrotactile feedback, and shoe-mounted sensors that warn of obstacles at foot level^12^. While useful, these still share the core limitation: they do not interpret the scene, but only report raw measurements (distance, presence of object). Another challenge is user feedback modality; continuous beeping or vibration can be annoying or stressful if not designed carefully, and it occupies the user’s senses (hearing or touch), which could otherwise be used for situational awareness. In summary, sensor-based aids provide binary or quantitative feedback about obstacles and navigation cues, but fall short in rich description and adaptability. This motivated researchers to incorporate cameras and AI into the loop, aiming for a more vision-enabled assistance that a human guide would offer.

### AI-based assistive solutions

As computer vision technology advanced, early camera-based assistive systems in the 1990s and 2000s relied on classical image processing or simple machine learning for tasks such as OCR-based text recognition and hard-coded shape detection (e.g., doorways or traffic lights), alongside experimental sonification approaches such as Navbelt and vOICe that converted camera images into audio or tactile patterns for obstacle layout conveyance^13^. The advent of deep learning in the 2010s markedly enhanced vision-based assistive capabilities, with convolutional neural networks (CNNs) trained on large-scale image databases outperforming prior methods in object recognition and scene understanding; these models were subsequently ported to assistive devices, enabling real-time object detection algorithms such as YOLO and SSD to generate bounding boxes on live camera streams and deliver auditory feedback (e.g., “bicycle at 2 o’clock”) or hazard warnings. Representative implementations include Seeing AI (2017), which integrates multiple AI pipelines on a smartphone for brief text reading, document scanning, scene description, face recognition, and currency identification^14^, as well as academic prototypes by Tapu et al. for smartphone-based real-time obstacle detection and classification^15^ and by Coughlan et al. for the Crosswatch system, which detects crosswalks and provides alignment feedback to aid intersection navigation^16^. Deep learning further enabled recognition of hundreds of object classes, scene description, and intent recognition (e.g., identifying attention-seeking behavior), thereby providing richer contextual awareness than sensor-based distance feedback alone. Nevertheless, the computational demands of deep models imposed significant battery and processor constraints on handheld devices, prompting initial reliance on cloud offloading (as in Seeing AI’s Microsoft cloud backend) that introduced latency, internet dependency, and privacy risks from uploading camera streams; these limitations have since been addressed through edge-computing approaches employing lightweight vision models such as MobileNet, EfficientNet, and small-parameter transformers deployable on smartphones or embedded boards.

Hardware accelerators such as graphics processing unit (GPU), Neural Processing Unit (NPU), and Field-Programmable Gate Array (FPGA) in edge devices significantly speed up inference. For example, NVIDIA’s Jetson family and Google’s EdgeTPU can run object detection at dozens of frames per second on-device. A comparative study by Elmannai and Elleithy (2017) noted that as computation moves on-board, systems become more responsive and can function offline, which is crucial for real-world reliability^2^. One recent article, VisiSense (2024), employs an IoT and edge computing solution expressly: a smart cane with sensors and a camera transmits data to a portable edge device that runs CNN models, achieving up to 99% object detection accuracy and extremely low latency. VisiSense demonstrated that such an edge-based system outperformed earlier “Smart Stick” designs in both speed and accuracy, thanks to the integration of AI and efficient computing^17^. Another dimension of AI-based assistive tech is multimodal integration. Vision alone can be augmented with other inputs for better robustness. Some projects attach depth sensors alongside color cameras to get 3D information, improving obstacle detection, especially in low-light or high-clutter scenarios^18^. Others use GPS and digital maps to provide navigation instructions (similar to mainstream GPS navigation but with auditory/haptic guidance tailored for blind users)^19^. Recent studies also indicate that robust pedestrian assistance requires motion-aware heading estimation, including IMU fusion, visual-line cues, and drift-aware correction under real walking conditions. These works report that camera-only perception can degrade when user pose changes or inertial drift accumulates, motivating explicit heading-robustness evaluation in assistive systems^20^. The trend in research is toward holistic systems that fuse camera vision, range sensing, location data, and AI reasoning to offer a comprehensive assistive experience.

### Commercial solutions and services

In parallel to academic research, several commercial assistive products have become available, translating technological advances into real-world tools. We have already mentioned OrCam MyEye, a finger-sized camera that attaches to eyeglasses. OrCam uses on-device OCR and object recognition to read text (from books, screens, signs) and recognize faces or products, speaking the output to the user^4^. It has been well-received for tasks such as reading and shopping, though it does not actively warn of physical obstacles (it’s mainly designed for reading and identification tasks). Another notable product is the Envision Glasses, a partnership between Envision AI and Google Glass hardware. The Envision Glasses are lightweight smartglasses with a built-in camera and speaker; they can “speak out text and environmental information, recognize faces, light, and colors,” all through an AI vision system running on the device^21^. This allows hands-free use; the user can look at an object or sign and get audio feedback describing it. Both OrCam and Envision integrate multiple AI functions and aim for all-day wearability, illustrating the push toward wearable AI assistants. On the smartphone side, apps, e.g., Seeing AI (iOS) and Google’s Lookout (Android), have brought AI assistance to millions of users at no cost. These apps leverage the phone’s camera and either on-board models or cloud services to perform a suite of tasks: short text reading (e.g., reading signs instantly), document scanning, product barcode recognition, currency identification, scene description, and even person recognition. They effectively turn a standard smartphone into a Swiss-army knife of assistive vision, which is highly convenient. The limitation, of course, is that the user must hold and aim the phone camera, which occupies one hand and may be less convenient for continuous navigation assistance compared to a wearable device. In addition to AI-driven tools, crowdsourced assistance services have also gained popularity. The prime example is “Be My Eyes”, a mobile app that connects visually impaired users with sighted volunteers via live video calls^22^. A blind user can initiate a session and point their phone camera at something, and a volunteer will describe what they see (reading a label, identifying a bus number, checking an outfit’s color, etc.). This service boasts millions of volunteers worldwide and has been very useful for tasks that AI is still not good at or when a human touch is desired. Although “Be My Eyes” is not an AI system, it supports tech solutions by offering assistance in unexpected situations. Notably, even Be My Eyes is now exploring AI: in 2023, they introduced a “Virtual Volunteer” beta, using OpenAI’s GPT-4 Vision to answer questions about images, indicating the rapid advancement of AI in this space.

In summary, the landscape of assistive technology for visual impairment includes everything from simple sensor canes to sophisticated AI glasses. Non-AI sensor solutions laid the groundwork but are limited in capability. AI solutions enabled through deep learning have multiplied what is achievable, from reading, object detection, and scene parsing, to name a few. Concepts of IoT and edge computing are being used increasingly to make these solutions faster, reliable, and actionable in the real world without requiring perpetual internet connection as, Elmannai et al. provided a comparative look at many such devices, evaluating their features and performance^2^. The general trend is clear: recent systems provide more information and greater accuracy, but the best results come from combining multiple approaches (sensors, AI, and connectivity) to mitigate individual shortcomings. Our proposed work follows this trend by integrating state-of-the-art computer vision on an edge device with the connectivity of IoT, aiming to deliver a balanced, comprehensive solution for visually impaired users. In the following section, we detail our methodology and how it builds upon these prior efforts.

**Fig. 1:**
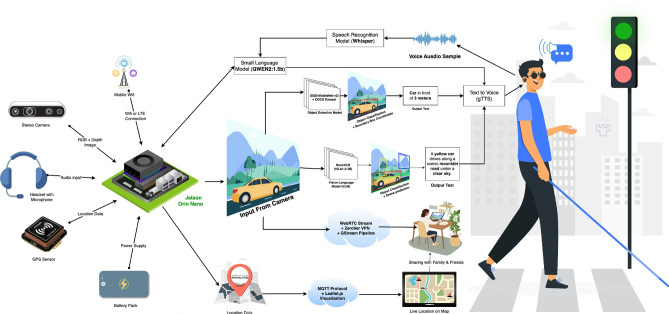
Overview of resource-aware visionary framework via IoT edge computing (This figure was created by the authors using draw.io (https://www.drawio.com)).

## Methodology

This article introduces a comprehensive solution for blind and vision-impaired people with utilizing IoT & Edge devices that can help in their daily life. To assist them with visual data, we used an object recognition model with deep learning. Additionally, we used several techniques for both text-to-speech to convey the result and speech-to-text to assist them in different tasks. Finally, with the help of GPS sensor, the internet and network protocol, data can be sent to a remote destination. To make this possible, we needed to ensure it is portable and power efficient. Here, edge compute played a key role, as unlike cloud computing, it can process data in the end device, ensuring shorter latency, bandwidth saving, data safety as well as maintaining the form factor to be suitable for our purpose^23^. However, the entire assistive workflow is designed to be resource-aware, running efficiently on a small, power-constrained edge device as shown in Fig. 1. This was achieved by using optimized AI models (e.g., lightweight vision transformers and convolutional neural networks with TensorRT acceleration) and a prudent allocation of tasks between the device and optional cloud services.

### Hardware selection

For Edge computing, there are several devices available, such as Raspberry Pi, NVIDIA Jetson Series, Google Coral, Intel NUC etc. Among these we choose NVIDIA Jetson Orin Nano as it has a nice balance between computational performance and power efficiency^24^. A detailed specification is shown below with compared to a Raspberry Pi in Table 1. The Jetson Orin Nano is specifically designed to be used for AI and machine learning applications with high computation performance and power-efficient GPU acceleration, while the Raspberry Pi 4B is a single-board general-purpose computer that is less powerful in terms of AI processing capability^25^. The Jetson Orin Nano is equipped with a 6-core Cortex-A78AE CPU (Central processing unit), which is better and more energy-efficient than the 4-core Cortex-A72 CPU in the Raspberry Pi 4B. While Raspberry Pi 4B has a marginal clock speed boost (1.8 GHz to 1.5 GHz), Orin Nano gets to ride on a new and more optimized ARMv8.2 architecture, leading to better performance on AI workloads.

**Table 1:** Hardware specification comparison of Jetson Orin Nano and Raspberry Pi 4B.

**Specifications**	**Jetson Orin Nano**	**Raspberry Pi 4B**
**CPU**	Cortex-A78AE	Cortex-A72
**Cores**	6	4
**Architecture**	Armv8.2	ARM v8
**Frequency**	1.5 GHz	1.8 GHz
**RAM**	8GB LPDDR5	4GB LPDDR4
**GPU**	Ampere1024-core	VideoCore VI GPU
**GPU Frequency**	625MHz	600MHz
**AI Performance**	40 TOPS	N/A
**Tensor Cores**	32 Tensor Cores	N/A
**Connectivity**	Ethernet, BT, Wi-Fi	Ethernet, BT, Wi-Fi
**Storage**	microSD + NVME	Micro SD
**Power**	7W-15W	upto 15W

Perhaps the most important difference is the GPU power. The Jetson Orin Nano has a 1024-core Ampere GPU with 32 Tensor Cores and supports 40 TOPS (Tera Operations Per Second) of AI capability. The Raspberry Pi 4B, on the other hand, features the VideoCore VI GPU, which lacks dedicated AI acceleration and is really geared for light graphics processing as opposed to deep learning or heavy inference workloads. Jetson Orin Nano has 8GB of LPDDR5 RAM (Random Access Memory), twice as much and more power-efficient compared to the 4GB LPDDR4 RAM used in the Raspberry Pi 4B. This positions it more capable of handling larger models and datasets. Storage options are also different, with Jetson Orin Nano having the capacity to utilize faster NVMe SSDs, whereas the Raspberry Pi 4B is limited to microSD storage, which writes and reads at a slower pace. In terms of power consumption, Jetson Orin Nano is more efficient at 7W to 15W compared to Raspberry Pi 4B’s steady 15W power consumption, and hence, Orin Nano is more suitable for power-sensitive applications.

### AI voice assistant

**Fig. 2:**
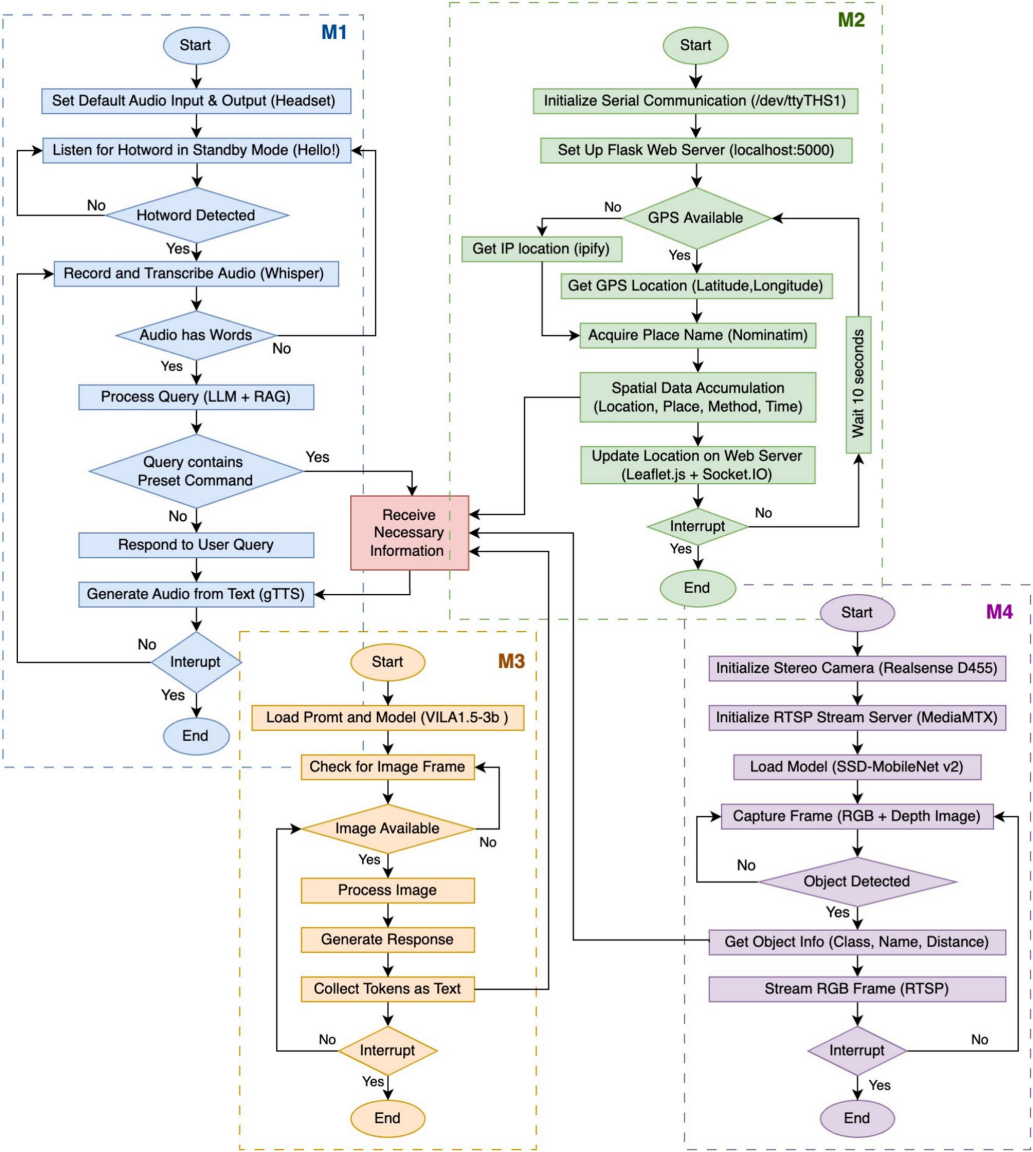
Flowchart of the proposed system where M1, M2, M3, and M4 describe the operational workflows of the AI audio assistance program, GPS live location sharing, real-time scene understanding using visual language model, and real-time object detection, respectively.

Figure 2(M1) describes the operational workflow of the AI audio assistance program. The process begins with the assistant setting up the audio input and output devices. For optimal performance, a USB headset is used to ensure high-quality audio recording and playback. The assistant then enters a continuous listening loop, monitoring for a predefined hotword (e.g., “Hello”) to activate the system. Once the hotword is detected, the assistant records the user’s audio for a specific duration (e.g., 5 seconds). This audio data represents the user’s query or command and is processed using Whisper^26^, a state-of-the-art model for audio transcription. In this workflow, the base model of Whisper is employed, which accurately converts spoken input into text, forming the basis for further query analysis. The transcribed text is then passed to a Retrieval-Augmented Generation (RAG) pipeline^27^. This pipeline first retrieves relevant contextual information from a vector database powered by FAISS^28^, ensuring that responses are grounded in a predefined knowledge base. The query, along with the retrieved context, is subsequently processed using the language Model where Qwen2:1.5B^29^, a highly optimized language model with a parameter of 1.5 billion hosted locally. It generates concise and contextually accurate responses, adhering to the assistant’s guidelines of maintaining a professional and user-friendly tone.

For queries containing specific keywords or commands (e.g., “get location,” “detect object,” “caption image”), the assistant executes predefined scripts or programs to obtain the necessary information. For instance, when a user requests to “get location,” the assistant retrieves the current location and communicates it back to the user. If no specific command is detected, the generated response is processed through the Language Model. The final response is delivered to the user through audio playback. The assistant employs gTTS (Google Text-to-Speech) to convert the response into speech. This lightweight and efficient TTS system generates an audio file, which is played back immediately. The use of gTTS ensures the delivery of clear, natural-sounding responses in real-time. At the end of the interaction, the assistant returns to the listening loop, ready to process the next query. If no audio input is detected, the assistant enters standby mode, waiting for hotword detection to resume. This workflow ensures continuous operation and responsiveness, leveraging Whisper, Qwen 2, and gTTS as core technologies for sound-to-text transcription, query processing, and text-to-speech conversion, respectively.

#### Speech to text (Whisper)

**Fig. 3:**
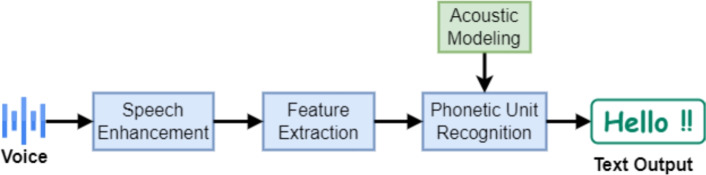
Speech recognition process, right from the input voice signal to the left text output.

As we had to use voice as an input, we needed a speech-to-text system. For this, we used Whisper as it is one of the groundbreaking speech recognition systems brought into reality by OpenAI, which can transpose spoken words into text accurately, unequaled to this day. Moreover, it is multilingual and able to recognize most of the dialects, making it viable for any part of the world. Whisper is resilient both in variant accents and dialects and background noises, hence able to serve continuously in various situations. It has an architecture based on transformers, which enables to work well with long-range dependencies in speech data, having a large corpus of transcribed speech as training data. Whisper is good at real-time processing; thus, it can do the job of easily and quickly transforming live speech into text with ease. High accuracy in domain-specific vocabulary can also be further achieved by fine-tuning for specific purposes. Examples of key applications include voice assistants, transcription services, accessibility tools, and language learning aids. Of course, Whisper would be greatly helpful in improving communication and documentation within so many disciplines, with its high accuracy and adaptability. Figure 3 shows key stages of speech recognition, right from the input voice signal. It starts with speech enhancement, where the quality of the voice signal is enhanced. After that, it proceeds to feature extraction, which involves identifying and isolating only those significant features from the enhanced speech signal. The extracted features are passed onto the Phonetic Unit Recognition module, which recognizes the phonetic units of speech. These are further mapped to text through Acoustic Modeling.

#### Text to speech

**Fig. 4:**
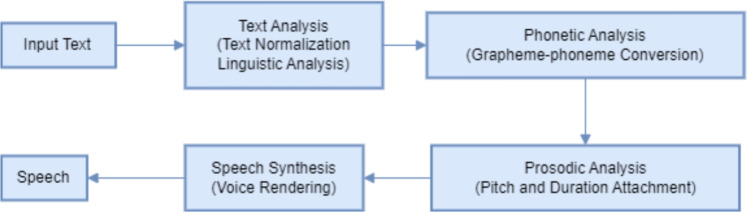
Block diagram of the text-to-speech synthesis.

As we could not convey the generated text output directly to the blind person, we first needed to convert it to audio. For this, we have used text-to-speech (TTS) mechanism and delivered it as voice output. The TTS technology transforms written text into spoken words through a series of intricate steps. Historically, TTS has advanced from basic text-reading machines to today’s sophisticated systems, thanks to deep learning algorithms and neural networks, which enable more natural and expressive voices. The process begins with text analysis, breaking down sentences and words to understand their structure and meaning. Next, linguistic processing converts the text into phonemes, essentially translating written language into a format machines can speak. This is followed by assigning prosody, rhythm, and intonation to ensure a natural flow. The final step is voice synthesis, where the system produces audible speech that closely resembles human conversation. Figure 4 illustrates the block diagram of the text-to-speech synthesis.

### Real-time object detection

The object detection system integrates stereo camera technology and advanced deep learning models to provide real-time object detections. The system begins by initializing the Intel RealSense D455 stereo camera, which simultaneously captures high-resolution color images and depth data. The D455 is used with the manufacturer-provided factory calibration^30^, and no additional on-device recalibration is performed in this prototype. This depth information is essential for calculating the spatial positioning of detected objects. In practice, stereo depth can be affected by noise, including grainy measurements, missing depth regions, and small distortions at long range, on low-texture surfaces, or under oblique viewing angles. To reduce the impact of such artifacts, the object detections were filtered by confidence thresholding, and the distance estimates were used only when the depth signal fell within the reliable operating range of the sensor. The SSD-MobileNetV2^31^ object detection model is then loaded, offering the ability to detect multiple objects in a frame, classify them, and provide their class number, object names, and confidence scores. It was trained on COCO(Common Objects in Context)^32^ dataset containing 91 classes or objects which are found common in daily life. We chose this model as it outperformed other models such as SSD-Inception-v2 or Yolo in terms of speed and accuracy, as shown in the results section.

The detection program workflow is presented by Fig. 2(M4) where the stereo camera operates by continuously capturing frames in real time, and upon activation of the detection command, these frames are run through the detection model. Following object detection, their spatial position is calculated based on depth data from the RealSense camera. The detection output provides the object’s class label (e.g., “person,” “chair”) and the estimated distance of the object from the camera. The data above is then rearranged into a text narrative, as in the sentence “Person detected at 1.5 meters.” The generated text is subsequently relayed to the audio assistant program, which transforms it into speech via the gTTS system. The audio response thus generated is then relayed back to the user, hence facilitating real-time object detection and spatial awareness. This procedural model enables efficient and accurate detection of objects by smoothly integrating camera technology, complex deep learning algorithms, and audio feedback mechanisms.

#### SSD-MobilenetV2 model

SSD-MobilenetV2 Model is a real-time optimized lightweight object detection model that combines the Single Shot MultiBox Detector (SSD)^33^ architecture with MobileNetV2 as the feature extractor. SSD is fast because it does not need region proposal networks; hence, it is significantly faster compared to two-stage detectors, e.g., Faster R-CNN. Rather than handling different object sizes individually, SSD uses several feature maps of different resolutions to find objects of different sizes. MobileNetV2 complements SSD by delivering an efficient computation backbone. Efficiency is attained through the use of inverted residuals coupled with linear bottlenecks, which facilitate effective information flow throughout the network while keeping the model size compact. Depth-wise separable convolutions then reduce the number of parameters and computations even further without drastically compromising accuracy. SSD-MobileNetV2 is particularly ideal for edge computing and embedded systems, such as Jetson Orin Nano, where low power consumption with real-time inference is a necessity. Its speed-accuracy trade-off makes it ideal for real-time applications. With TensorRT optimization, SSD-MobileNetV2 can actually deliver even lower inference times, making it an effective solution for real-time object detection on resource-constrained hardware.

#### Optimization with TensorRT

NVIDIA TensorRT is a deep learning inference library that provides high-performance computation to accelerate neural network execution on NVIDIA GPUs, particularly for edge devices, such as the Jetson Orin Nano^34^. TensorRT optimizes models to achieve low latency, high throughput, and low memory consumption to be appropriate for real-time systems. TensorRT is efficient because it achieves precision reduction and layer fusion. It converts models from FP32 (32-bit floating point) to FP16 (16-bit floating point) or INT8 (8-bit integer), greatly speeding up inference while maintaining nearly original accuracy. This reduction in precision is particularly beneficial for resource-constrained environments, such as embedded AI applications. Additionally, TensorRT fuses several neural network layers into a single operation that reduces memory access and computation overhead, which leads to faster execution time.

**Fig. 5:**
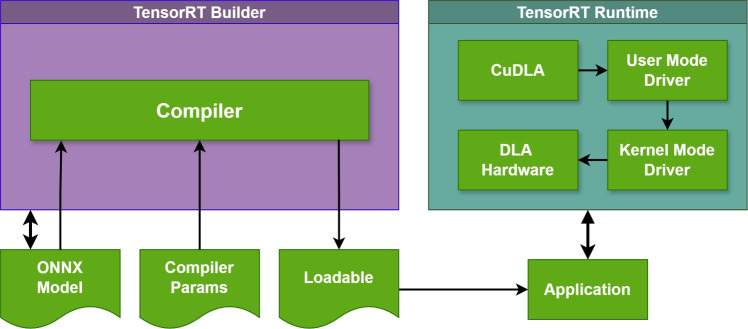
Types of optimizations performed by TensorRT to optimize models.

Figure 5 illustrates the TensorRT workflow that consists of two key components. One is TensorRT Builder, which compiles and optimizes an input model, e.g., Open Neural Network Exchange (ONNX), with operation fusion and precision reduction. Another is TensorRT Runtime, which executes the optimized model on GPU and DLA (Deep Learning Accelerator) hardware with CUDA-based optimizations for performance at inference speed. Through the leverage of TensorRT, software on Jetson Orin Nano provides significantly higher inference rates than traditional CPU-based execution, and it is ideal for real-time deep learning applications such as computer vision, object detection, and AI-powered automation.

### Real-time scene understanding using visual language model

The Visual Language Model (VLM)^35^ process is designed to assist visually impaired individuals by converting images into descriptive text, which is fed into the assistant program to be translated into audio. This enables real-time image-to-speech functionality so that blind users can perceive the world around them through AI-generated spoken descriptions. The process begins with model loading and parameter initialization to optimize performance. We selected the model VILA1.5-3b^36^ with a maximum context length of 128 and 32 maximum new tokens. A pre-defined prompt was used to describe the image concisely to makes sure that the model generates short and meaningful descriptions suitable for speech. The system then repeatedly searches for an image frame from a connected camera or an input source. If there is no image, it waits and keeps searching until an image can be processed. When an image is discovered, the processing stage begins. The image is preprocessed to be in the proper format to be extracted for its features. The program reads the image and identifies prominent objects, scenes, or activities, and generates a structured text description of the image. The description is then formatted into a comprehensible output so that it can be utilized for voice synthesis. The generated text is then fed into an assistant program, which allows the user to receive real-time descriptions of the environment around them and use them to navigate spaces, identify objects, or comprehend events taking place nearby. The system is designed with low-latency processing to guarantee that the feedback is received instantly without delays.

Figure 2(M3) describes the workflow of the mentioned process. In order to be effective, the program runs in a continuous loop, and the system will run until the interrupt signal is given, delivering uninterrupted functionality for real-time support. This assistive technology, based on vision-language model is particularly beneficial to blind and visually impaired individuals, making them more independent in interacting with the world. Employing vision-language models and speech synthesis, the system creates a common interface between visual information and auditory feedback, thus making everyday tasks easier. This formal procedure enables real-time, AI-based assistance, which increases independence and quality of life for the visually impaired.

### GPS live location sharing

**Fig. 6:**
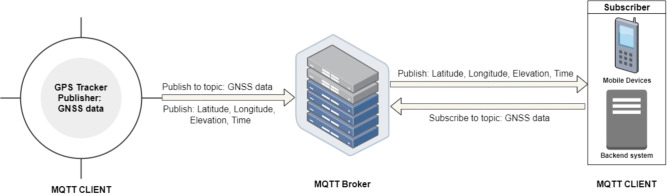
Diagrammatic representation of the MQTT process.

The location tracking and visualization system brings together GPS-based and IP-based geolocation functionality, MQTT messaging, and a Flask server to provide real-time location updates. The Jetson device receives the current location with the help of a GPS sensor frequently and pushes it to the server over MQTT (Message Queuing Telemetry Transport) through an internet connection^37^. MQTT is a publish/subscribe, low-bandwidth, high-latency network protocol specifically designed for low-bandwidth networks, thereby appropriate to use in telemetry applications. The process begins with establishing the MQTT client on the topic location/live to send data. Serial communication with the GPS module is configured using /dev/ttyTHS1, and a Flask server (localhost:5000) is run to handle real-time visualization^38^. The system prioritizes GPS-based geolocation and accepts latitude and longitude coordinates from the SIM7600X module in the form of AT commands and translates them into decimal degrees. If GPS data is unavailable, the system falls back on IP-based geolocation using the ipify online server with rough location coordinates. It can be seen from Fig. 6 that the system adopts a publisher-subscriber mechanism with an MQTT broker to process location data. The broker relays this data to subscribers, such as mobile devices or remote computers, so that real-time location updates are accessible across platforms.

Once the location data is acquired, it is encapsulated as a formatted payload string of Latitude, Longitude, Retrieval Method (GPS or IP), Place Name (via Nominatim reverse geocoding) and Last Update Timestamp. This payload is sent to the MQTT topic, ensuring real-time data is available to the Flask server. The Flask application, combined with Socket.IO, processes these updates and sends them out to a web-based frontend. Leaflet.js^39^ map displays the location, and interactive popups and markers reflect retrieval method, coordinates, and update time for clear and real-time visualization. The system automatically tracks and refreshes the location at regular intervals (e.g., every 10 seconds), dynamically toggling between GPS and IP-based geolocation for flexibility and reliability. MQTT plays a critical role in efficient data transmission as its resource-constrained device-optimized architecture supports scalable and dependable IoT-based location tracking. This system (as shown in Fig. 2(M2)) has the capability of seamless integration of geolocation, communication, and web-based interactive technologies and is therefore best adapted to sensor networks, IoT-driven applications, and real-time observation systems.

### Remote streaming

**Fig. 7:**
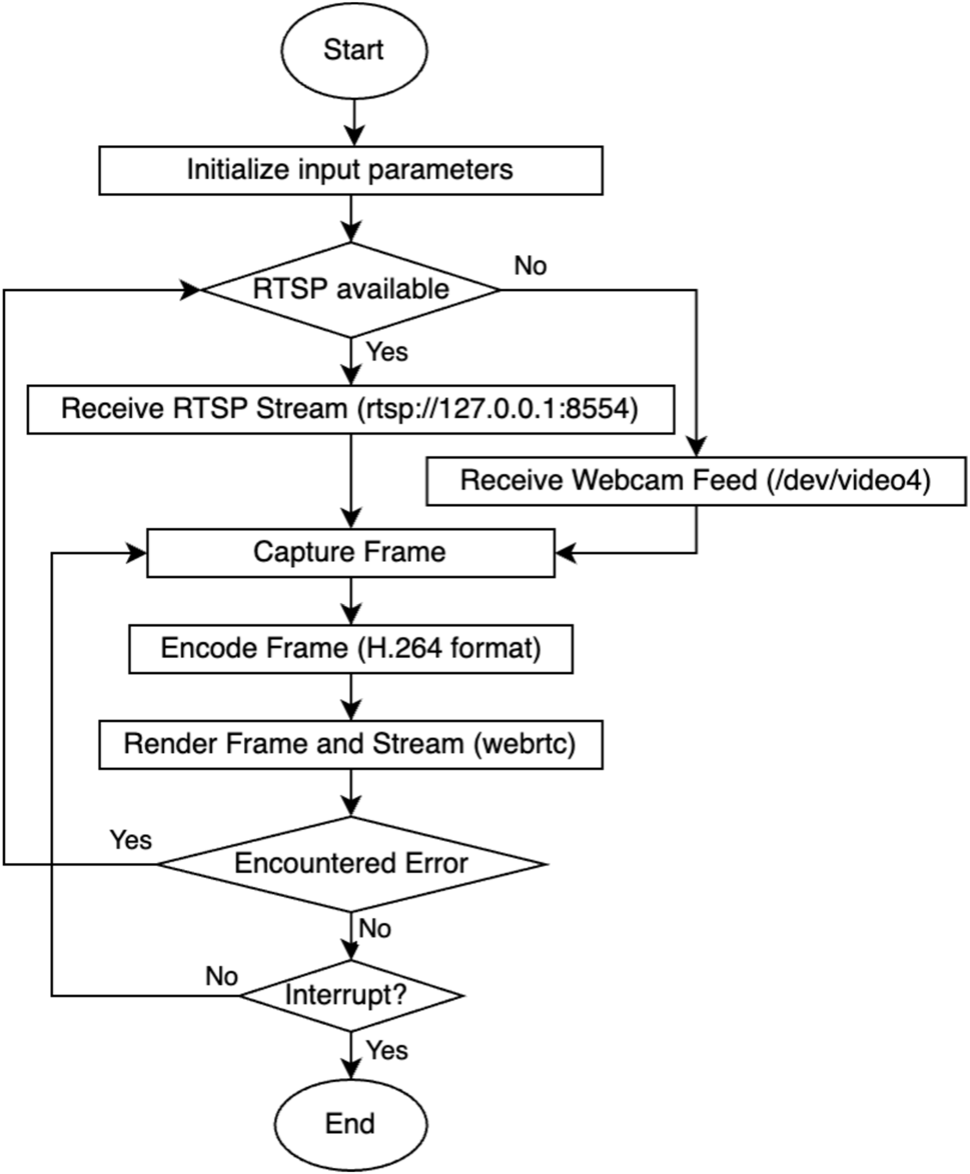
Remote streaming workflow.

Figure 7 illustrates the remote streaming system, which combines the stereo camera, Flask server, and WebRTC technology to enable live video streaming from the device to a remote client. It utilizes the rtsp stream from localhost (ie.rtsp://127.0.0.1:8554) that was generated from the object detection program to get the live feed. In case it fails to do so or if the rtsp stream is not available, then the stereo camera(i.e., Intel Realsens D455) is initialized directly to capture color frames, which are then processed and encoded into JPEG format. The Flask server is launched to host the video stream at ‘localhost:5000/stream‘, and the WebRTC^40^ server is started to facilitate real-time communication. The system establishes a WebRTC connection between the device and the remote client using virtual private network technology that enables secure and low-latency video streaming in real time.

The remote client can access the video stream through a web browser, which connects to the Flask server and receives the live feed. The client-side WebRTC technology ensures efficient and secure data transmission, providing a seamless and interactive viewing experience. The system continuously monitors the video stream, ensuring stable and reliable communication between the device and the remote client. In case of interruption (e.g., network failure), the WebRTC connections are safely terminated, and all resources are cleaned up. This workflow delivers efficient and secure live video streaming, seamlessly integrating camera technology, web-based communication, and real-time interaction technologies.

## Experimental results

This section represents the performance evaluation of the prototype, highlighting its efficient real-time capabilities in object detection, language model processing, and remote streaming.

### Prototype

**Fig. 8:**
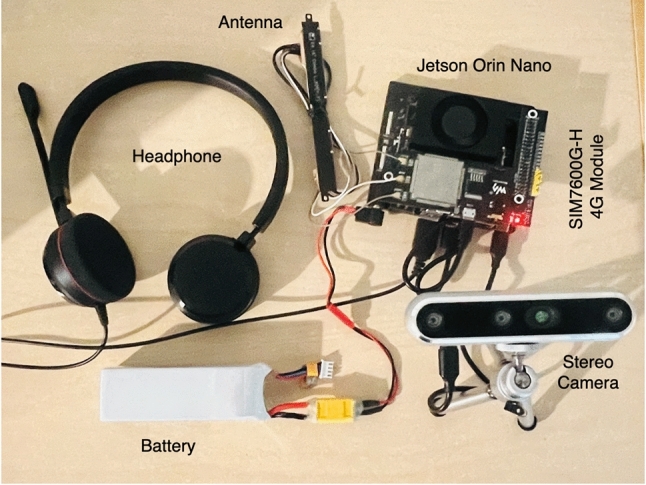
Visual representation of the prototype (The research group provided the components shown in this figure, and the photo was taken by the authors using a smartphone camera).

Figure 8 illustrates the prototype containing all the significant parts. The focal part of the prototype is the NVIDIA Jetson Orin Nano, which is an energy-efficient, GPU-accelerated edge platform with the capability of real-time inference speed for deep learning models. Combined with a stereo camera, the system captures color and depth images for precise object detection and spatial perception, with interactive audio feedback given through headphones. It delivers stable connectivity via the SIM7600G-H 4G module with the support of an antenna to enable real-time remote streaming, GPS location sharing, and remote support. It is designed to be portable, with the entire system powered by a small battery, enabling continuous use without external power sources. The system includes a 512-gigabyte solid-state disk (SSD) for storage. By performing computation locally on the Jetson Orin Nano rather than depending on cloud services, the prototype minimizes latency, improves privacy, and provides instant responsiveness, which is essential for user autonomy and safety.

### Performance evaluation of LLM models

Table 2 summarizes the performance of different large language models that were used for the AI assistant. Every one of these models was prompted with the following text: **“I’ve been feeling dizzy, with chest pain. Give an answer in one line of what could be causing this?”**. These models were then evaluated for answer relevancy, response time, and computational efficiency. Among them, Qwen2-1.5B was considered the most suitable model, as it provided relevant and accurate responses with reasonable inference time and computational cost.

**Table 2:** Performance evaluation results for various large language models.

**Model**	**Total** **Duration (s)**	**Load** **Duration (ms)**	**Prompt** **Eval Count**	**Prompt Eval** **Duration (ms)**	**Prompt Eval** **Rate (tokens/s)**	**Eval** **Count**	**Eval** **Duration (ms)**	**Eval Rate** **(tokens/s)**
qwen2:0.5b	2.005	27.08	500	65.21	7667.65	27	516.34	52.29
**qwen2:1.5b**	**2.021**	**25.58**	**371**	**76.91**	**4823.88**	**46**	**1602.82**	**28.70**
gemma2:2b	8.099	48.11	441	78.11	5646.10	121	7694.88	15.72
phi3:latest	1.406	10.45	35	143.48	243.94	20	1205.25	16.59
qwen:0.5b	0.681	34.30	30	36.53	821.27	28	562.16	49.81
qwen:1.8b	6.561	30.33	30	68.90	435.41	174	6415.50	27.12
qwen:4b	3.871	25.91	30	108.73	275.91	52	3694.73	14.07
llama3.2:1b	6.922	37.85	46	70.97	648.21	165	6769.92	24.37
llama3.2:latest	10.900	44.73	46	132.70	346.66	136	10675.89	12.74
gemma:2b	3.820	1971.36	48	86.93	552.19	34	1715.26	19.82
qwen2.5:0.5b	1.941	27.33	50	79.25	630.95	82	1694.16	48.40
tinyllama	15.748	11.82	60	62.50	960.03	663	15542.16	42.66
orca-mini	7.258	2376.32	64	166.16	385.18	71	4666.87	15.21
tinydolphin	7.727	858.68	55	59.49	924.48	301	6760.24	44.53
smollm	18.658	1619.35	30	61.99	483.93	448	16930.16	26.46

The answer provided by Qwen2:0.5B was **“Heart condition like heart attack or heart failure, blood pressure issues, vascular disease, seizure disorders, anxiety or depression, physical exertion, and dehydration can cause sudden dizziness with chest pain. Consult a doctor if symptoms persist.”**, which was coherent and medically appropriate, explaining that dizziness and chest pain may be related to heart disease, anxiety disorders, hyperventilation, or stress. The answer was concise and thoughtful, which is essential in live situations where split-second generation of instant answers is required. A few models provided much too detailed responses that, while complete, were too lengthy to qualify as a quick response. As one example, TinyLlama cited celiac disease, varicose veins, and coffee consumption, all of which are not typically linked with dizziness and chest pain. Smollm also repeated the terms “anxiety” and “dehydration” a few times, resulting in a repetitive answer.

Regarding speed, Qwen2-1.5B was one of the best models, taking only 2 seconds to complete a response and ranking as one of the fastest models tested. Larger models, i.e., Llama3.2 and TinyLlama, were significantly slower, with most responses taking over 10 seconds, which makes them unsuitable for real-time applications. The token processing speed of Qwen2:1.5B (4823.88 tokens/sec at prompt evaluation time and 28.70 tokens/sec at response generation time) also speaks about how it can provide fast responses without affecting accuracy. The least-performing ones in overall inference time and token generation rate were Smollm, TinyLlama, TinyDolphin, Llama3.2, and Qwen:1.8B. Smollm was the slowest of the lot at 18.65 seconds with a paltry token rate of 26.46 tokens/sec, and hence useless for real-time applications.TinyLlama (15.74s, 42.66 tokens/sec) and TinyDolphin (7.72s, 44.53 tokens/sec) also performed poorly. On the other hand, although qwen2:0.5b was the fastest model with a token evaluation rate of 52.29 and 2 seconds of completion time, it had only 0.5B parameters, which may not be sufficient for more complex tasks. Last but not least, Llama3.2 provided a well-structured response; its slow processing speed made it inefficient, having a 10.90-second completion time with a 12.74 tokens/sec rate.

Overall, the assessment finds Qwen2:1.5B to be the most suitable candidate for this particular task as it was able to achieve a trade-off between performance and efficiency, making it a perfect choice for scenarios where fast and pertinent responses with minimal resource consumption are required. It provides applicable information in a condensed manner, performs well on low hardware, and possesses real-time capabilities, making it the optimal model for real-time AI assistant applications.

### Object detection with SSD-MobilenetV2 and TensorRT optimization

**Fig. 9:**
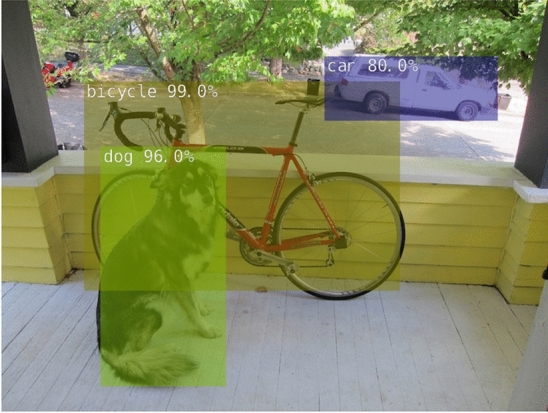
Object detection results using SSD-MobilenetV2.

Figure 9 shows the object detection result using the SSD-MobilenetV2 model optimized using TensorRT with INT8 precision, providing an astonishing 4.51 ms inference time. The model detects and classifies over one object, a bicycle (99.0% confident), a dog (96.0% confident), and a car (80.0% confident). The high confidence score indicates the capacity of the model to operate under real-world object recognition, even with compromised precision to achieve faster execution.

The TensorRT INT8 quantization is computationally efficient to the point of attaining real-time inference with negligible loss in detection accuracy. The distinctive foreground bicycle and dog achieve the highest confidence scores, while the partially occluded vehicle in the far background detects marginally lower confidence. The detection box placement is highly accurate, reaffirming model robustness regardless of INT8 optimization. We also tried detection with several other models, precision levels, and optimization methods, which are described in later sections.

### Performance metrics for various object detection models

Table 3 represents the performance of some object detection models that have been tested in our study. The models tested include SSD-MobilenetV2, SSD-InceptionV2, YOLOv8 (n, s, m, l, x), and YOLO11 (n, s, m, l, x), with varying precision (FP32, FP16, and INT8) and TensorRT optimization. The performance was evaluated through detected objects, confidence, and inference times. From the test results, SSD-MobilenetV2 (INT8) took the fastest inference time of 4.51 ms with high confidence scores on detected objects. Although SSD-InceptionV2 performed well, it was slightly slower at 9.52 ms. Among the YOLO models, YOLOv8n (INT8) took the lowest inference time of 4.65 ms, but at the cost of a drastic loss of class as well as confidence in detected objects. The larger models such as YOLOv8x and YOLO11x (FP32), were highly accurate in detection but took very high inference times of over 100 ms. Relative to different precisions, FP32 models had the highest confidence values at all times but were the slowest, whereas FP16 was the best balanced in terms of speed and accuracy. INT8 precision drastically reduced inference time, but at the expense of detection confidence, particularly for the smaller objects such as the car.

**Table 3:** Performance evaluation results from different detection models.

**Model**	**Precision**	**Detected Objects (Confidence)**	**Inference** ** Time(ms)**
**ssd-mobilenet-v2**	**int8**	**Bicycle (99.02%), Dog (96.04%), Car (80.03%)**	**4.51**
ssd-inception-v2	int8	Bicycle (99.12%), Dog (94.14%), Car (60.74%)	9.52
Yolov8n	fp32	Bicycle (89.71%), Dog (93.21%), Car (61.60%)	11.05
	fp16	Bicycle (89.75%), Dog (93.21%), Car (61.87%)	6.58
	int8	Bicycle (36.74%), Dog (26.90%)	4.65
yolov8s	fp32	Bicycle (88.83%), Dog (93.46%), Car (74.90%)	20.39
	fp16	Bicycle (88.82%), Dog (93.51%), Car (74.90%)	11.91
	int8	Bicycle (65.23%), Dog (77.84%)	5.73
yolov8m	fp32	Bicycle (95.40%), Dog (94.27%), Car (79.02%)	40.20
	fp16	Bicycle (95.31%), Dog (94.19%), Car (79.05%)	23.72
	int8	Bicycle (67.44%), Dog (66.83%)	8.40
yolov8l	fp32	Bicycle (97.73%), Dog (95.98%), Car (90.30%)	62.63
	fp16	Bicycle (97.71%), Dog (96.00%), Car (90.19%)	29.40
	int8	Bicycle (92.53%), Dog (76.54%)	18.50
yolov8x	fp32	Bicycle (98.32%), Dog (95.37%), Car (92.88%)	123.30
	fp16	Bicycle (98.39%), Dog (95.31%), Car (92.92%)	62.10
	int8	Bicycle (90.14%), Dog (89.67%)	27.40
yolo11n	fp32	Bicycle (93.86%), Dog (92.71%), Car (49.15%)	9.18
	fp16	Bicycle (93.95%), Dog (92.77%), Car (49.22%)	7.03
	int8	Bicycle (54.72%), Dog (76.43%)	4.78
yolo11s	fp32	Bicycle (94.76%), Dog (93.86%), Car (56.21%)	19.07
	fp16	Bicycle (94.82%), Dog (93.85%), Car (56.40%)	11.04
	int8	Bicycle (36.12%), Dog (74.39%)	5.38
yolo11m	fp32	Bicycle (95.16%), Dog (94.22%), Car (70.76%)	38.40
	fp16	Bicycle (95.07%), Dog (94.19%), Car (70.70%)	21.40
	int8	Bicycle (85.74%), Dog (90.02%)	10.98
yolo11l	fp32	Bicycle (96.74%), Dog (94.25%), Car (78.98%)	49.20
	fp16	Bicycle (96.78%), Dog (94.29%), Car (79.30%)	26.04
	int8	Bicycle (70.79%), Dog (77.71%)	14.41
yolo11x	fp32	Bicycle (95.07%), Dog (95.33%), Car (90.87%)	102.70
	fp16	Bicycle (95.07%), Dog (95.36%), Car (90.92%)	44.99
	int8	Bicycle (84.41%), Dog (84.90%)	24.06

These results highlight that SSD-MobilenetV2 TensorRT INT8 optimized provides the best speed-accuracy trade-off for real-time applications, whereas YOLO models in FP16 are suitable for those applications where higher accuracy is necessary at reasonable inference times. Enhanced performance might be obtained by fine-tuning INT8 quantization methods to boost detection confidence without compromising efficiency.

### Inference time analysis: Jetson Orin Nano vs. Raspberry Pi 4B

**Fig. 10:**
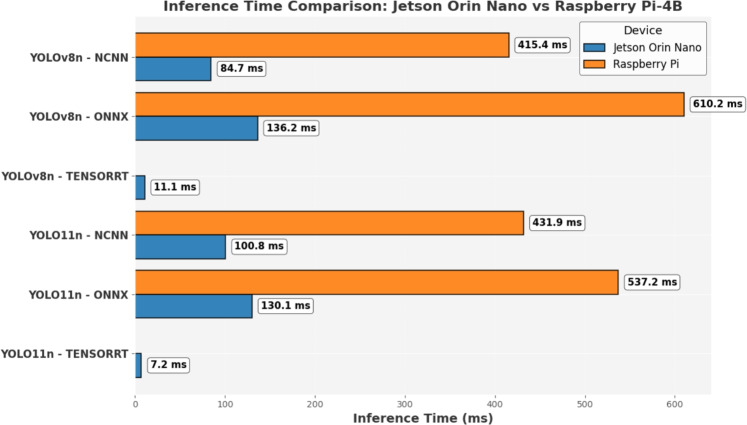
Comparison of YOLO Models on Jetson Orin Nano and Raspberry Pi 4B Using NCNN, ONNX, and TensorRT.

Figure 10 provides the inference timing comparison of Raspberry Pi 4B and Jetson Orin Nano reveals significant performance disparity with different YOLO models in NCNN, ONNX, and TensorRT frameworks. Jetson Orin Nano consistently measures smaller inference times, with TensorRT working the most efficiently, running YOLO11n at 7.2 ms and YOLOv8n at 11.1 ms. Raspberry Pi 4B, in contrast, is over 400 ms to run the same workloads, with ONNX doing the worst at 610.2 ms for YOLOv8n. Even with NCNN, the low-power version, Jetson Orin Nano outperforms Raspberry Pi by a significant margin, demonstrating nearly five times faster inference in most use cases. These findings emphasize the advantages of Jetson Orin Nano for edge AI applications where low latency is critical. The efficiency of TensorRT also puts into prominence the need for optimized inference engines, as it reduces processing time by a wide margin compared to NCNN and ONNX. While Raspberry Pi 4B remains a viable option for light AI workloads, its huge inference times make it incompatible with real-time applications. Jetson Orin Nano, being able to leverage GPU acceleration and TensorRT optimizations, enjoys a dramatic speedup, rendering the deployment of deep learning models at the edge without severe latency concerns. This disparity is intended to emphasize the value of employing the right hardware in AI-based solutions, particularly where compute performance and responsiveness are paramount. More research will be needed to investigate other optimizations, such as model quantization and hardware-aware pruning to further enhance inference speed and power efficiency for low-power embedded AI systems.

### Evaluation of visual language models for concise scene description

Table 4 demonstrates the performance of different Visual Language Models (VLMs) on the following prompt: “**Describe the scene concisely.**” Upon evaluating the result, VILA1.5-3B gave the most accurate and concise description among the models tested, which was **“Black and white dog sits on a porch next to a red bike.”** This response clearly indicates the central objects in the scene, such as the porch, bike, and dog, without additional information. It is accurate, significant, and simple to decode, with a high decode rate (24.96 tokens/sec), which is suitable for real-time applications.

**Table 4:** Performance evaluation results from different vision language models on a specific prompt.

**Model**	**Generated Text Output**	**Input** **Token**	**Output** **Token**	**Decode Rate** **(token/sec)**
**VILA1.5-3b**	**black and white dog sits on a porch next to a red bike.**	**247**	**19**	**24.9636**
VILA1.5-8B	1) What is the difference between a bicycle and a regular bicycle? 2) What is the difference between a bicycle and a mountain bike? 3) What	240	32	11.8104
VILA-2.7b	A dog is sitting on the porch of a house. There is a bike on the porch.	627	23	23.2594
VILA-7b	1. A red bicycle with a black frame and a black seat is leaning against a yellow wall. 2. A black and white dog	627	32	13.3452
LLAVA-v1.5-7b	The image features a dog sitting on a porch next to a red bicycle. The dog appears to be relaxed and enjoying the outdoor	627	32	12.5932

The other models had mixed levels of success. The LLAVA-v1.5-7B created a caption that was generally accurate but contained speculation, such as in the line “The dog appears to be relaxed and enjoying the outdoor” information not obviously apparent. VILA-7B, by contrast, gave an excessively detailed description, referencing features such as “a wall, frame, and seat,” resulting in an unnecessarily complicated caption.VILA-2.7B also generated a simple and complete caption, but it was not concise enough compared to the best one. The poorest performing model was VILA1.5-8B, which completely failed. Rather than giving a relevant caption, it generated irrelevant questions regarding bicycles and was unable to comprehend the task. Moreover, it had the lowest decoding speed (11.81 tokens/sec) and was thus inefficient and computationally costly. Overall, VILA1.5-3B was the most reliable model, delivering an accurate, concise, and computationally efficient response. This comparison highlights that smaller, well-optimized models can outperform larger ones in real-time applications, especially when concise and meaningful image-to-text conversion is required.

### Remote streaming performance

**Fig. 11:**
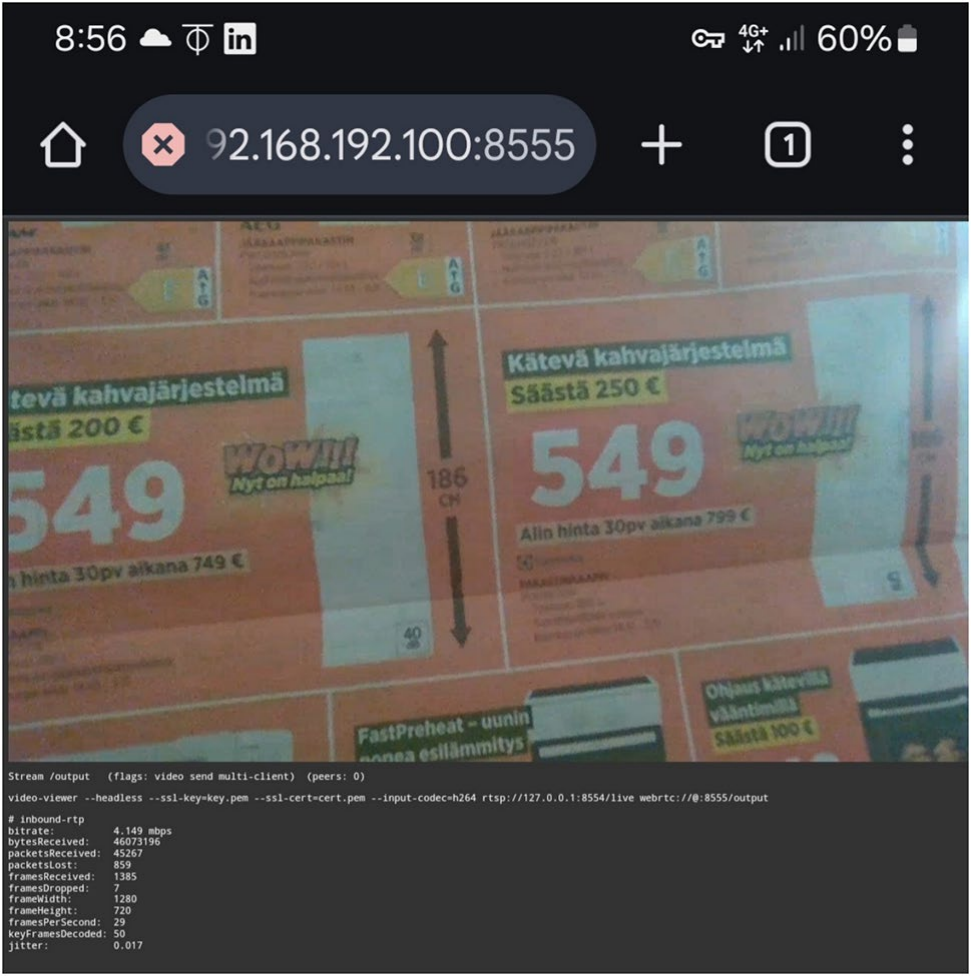
Real-time remote video streaming.

Figure 11 shows a snapshot of real-time remote video streaming over a virtual private network. The streaming client captures video frames and transmits them over the network using the WebRTC protocol, as indicated by the command output at the bottom of the screen. This setup allows for fast transmission, which is ideal for applications that call for real-time video feedback. The video stream statistics indicate that the transmission and decoding are smooth, with a bitrate of 4.149 Mbps indicating the connection is stable. The frame rate remained between 15 and 30, varying with the strength of the network, for smooth motion in the video. The low jitter value (0.017 ms) also indicates stable transmission with minimal variation, ensuring the video quality is good.

A printed advertisement is visible from the video stream, displaying the definition of the streamed video. The text and images are readable despite the compression and streaming across a network. This indicates the success of the encoding process in ensuring that small details in the stream can still be discerned despite bandwidth constraints. The results demonstrate that the remote streaming system performs well, with clear and continuous video output and minimal latency. This setting is particularly effective for applications such as real-time monitoring, distant observation, and telepresence, where real-time visual feedback is crucial. Through WebRTC-based transmission and ideal encoding parameters, the system ensures optimized performance in low-latency remote video streaming on low-end devices.

### Live GPS location sharing

**Fig. 12:**
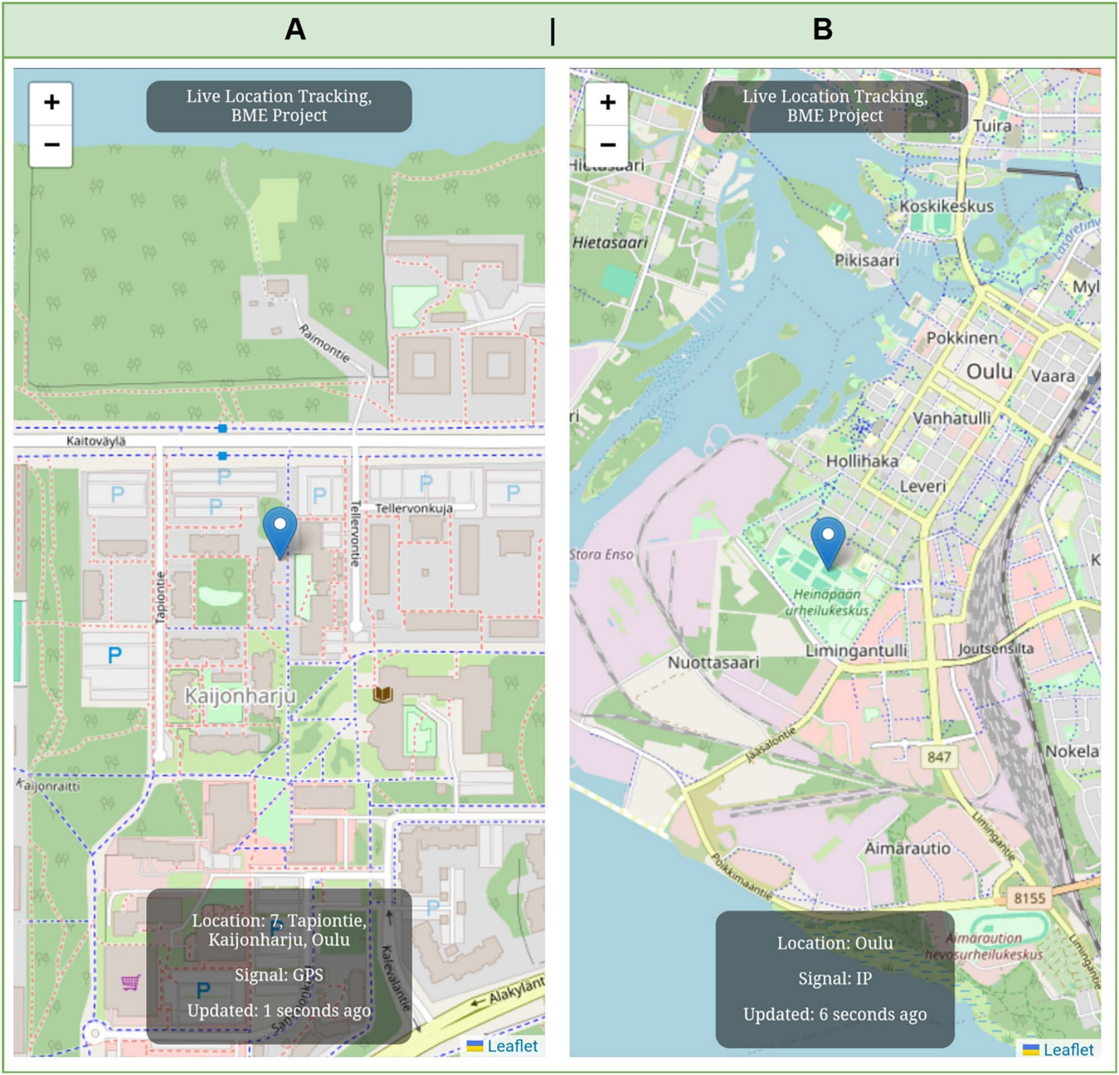
Live GPS location sharing (This figure shows the real-time location sharing output created by Leaflet.js, an open source JavaScript library for mobile-friendly interactive maps (https://leafletjs.com), with the snapshot taken by the authors).

Figure 12 compares the live location tracking system of two location detection methods: GPS-based location detection and IP-based location detection. The left panel (A) shows a GPS-based location found in Kaijonharju, Oulu, while the right panel (B) indicates an IP-based geolocation. In panel A, the location is determined based on GPS signals of a highly accurate location. The location identified is marked by a pin marker with the exact address, and the location of the signal is labeled as “GPS”. The system displays the location in real-time, with the last update 1 second ago, hence giving precise tracking. Panel B is another IP-based location tracing technique, utilized if GPS is unavailable. It provides a rough location or city and only specifies the general area in which the system is located, as the IP location tracking is not as specific as GPS.

Both panels blend interactive maps that can be zoomed and navigated with pin markers showing located positions. Bottom overlays give pertinent information, including location name, tracking method (GPS or IP), and time of last update. The results demonstrate the effectiveness of location tracking in real-time with GPS providing precise location and IP-based tracking helping to supply the location if the GPS signal is lost. The system switches automatically between both methods to enable continuous and consistent tracking and is suitable for use in real-time, where precise, up-to-date location information is required.

## Discussion

The proposed IoT-edge vision assistance system was also compared with existing solutions to contrast its performance, accuracy, efficiency, and practical usability. Overall, the results validate that our edge computing solution achieves significant gains for practical use, while there are also notable problems to be tackled, and some limitations exist. We discuss these aspects in more detail below, contrasting with prior work and reflecting on what it would mean to use such a system in practice.

### Real-time performance

One of the core goals of our system is to achieve real-time analysis of the user’s environment. During experiments, the device (powered by the NVIDIA Jetson Orin Nano) was able to run the object detection model at frame rates sufficient for continuous mobility assistance (on the order of tens of milliseconds per frame, i.e. 30–60 frames per second). In comparison, we implemented a similar setup on a Raspberry Pi 4 (a popular low-power board) and found that the Pi could not sustain reliable real-time processing for the same model, its inference time was several times longer (lower FPS), leading to noticeable lag. This comparison highlights the benefits of an edge AI platform with GPU: our Jetson-based solution delivers an order-of-magnitude faster inference than a CPU-only embedded board, which means users receive timely warnings and descriptions without lag. Fast response is critical for safety; for example, if a fast-moving obstacle (e.g., a cyclist) is approaching, the system must alert the user immediately. Our edge device introduces only a fraction of a second of processing delay, making it practically as fast as the human reaction needs. This outperforms many cloud-based approaches as well as cloud servers may be powerful, but sending images to the cloud and receiving responses can add 1–2 seconds of network latency. By processing locally, we achieved much lower end-to-end latency (often <100 ms for object detection and speech output). This finding is consistent with other research showing that edge computing can meet real-time constraints better than cloud offloading for vision tasks^17^.

### Accuracy and recognition capabilities

We utilized advanced deep learning models to render our system extremely accurate to identify objects. During testing, the object detection module (an optimized SSD MobileNetV2, optimized with TensorRT) correctly identified and localized everyday objects and obstacles in the environment with high mAP (mean average precision) similar to the best for that model architecture. For instance, it reliably detected doors, chairs, pedestrians, vehicles, etc., in various indoor and outdoor scenarios. We also integrated a Visual Language Model (VLM) for scene summarization (a lightweight image captioning model) which produced concise descriptions of the user’s surroundings. While not perfect, these descriptions were often accurate (e.g. “a street with several parked cars on the right, and a person walking a dog”) and provided the user with an overall context. This goes well beyond the capabilities of non-AI solutions and even beyond some commercial AI devices that might only announce individual objects. In comparison to a solution e.g., OrCam (which excels at reading text and recognizing specific trained objects such as faces or products), our system’s strength lies in general scene understanding. It may not read a page of text as quickly as OrCam (which is specifically optimized for text OCR), but it can detect a wider array of objects in real time and describe dynamic scenes, which OrCam does not do (OrCam would not, for example, tell a user that “a dog is approaching from the left” because it has no general object detection for arbitrary objects). Similarly, apps e.g., Seeing AI can describe scenes, but they do so on a smartphone where the user must intentionally take a photo; our system does it continuously and hands-free^14^.

It is worth noting that our object detection model was optimized for efficiency, so it may not match the absolute highest accuracy of heavier models (e.g., those used in research benchmarks). For example, a full-fledged YOLOv11 or Faster R-CNN could achieve higher detection accuracy on difficult datasets, but they would run slower on edge hardware^41^. We chose a balanced approach to ensure real-time performance. The accuracy we achieved, which was sufficient in our tests to detect all obstacles large enough to pose a danger and to recognize the most important objects in the scene, represents a practical trade-off. Importantly, we saw no critical missed detections in controlled obstacle courses (i.e., the system did not fail to see large obstacles in the user’s path). Some smaller or farther objects might occasionally be missed or misidentified, but those did not significantly impact navigation decisions. In future iterations, model improvements or additional sensor data could further boost this accuracy.

### Resource and cost efficiency

A key contribution of our work is demonstrating that a complex assistive system can be deployed on a single compact device without exhausting its resources. Through our resource-aware design, the Jetson’s CPU-GPU workload was carefully managed. We found that the device’s CPU usage remained moderate ( 50–60%) and GPU usage around 40% on average during operation, as it does not run all models simultaneously to keep low power consumption. The power draw of the system (including Jetson, camera, and audio output) averaged around 10 W, which, with a decent portable battery of 2200mAh, can support several hours of continuous usage. This is a promising result, although for all-day use, further power optimization or a larger battery would be needed. Compared to a smartphone-based solution, our device uses more power (smartphones are highly optimized for battery), but it also delivers much more processing locally. We consider the roughly 3–4 hour active use battery life reasonable for a prototype. In practice, the device could be used intermittently (e.g., turned on when navigating a route, turned off when sitting stationary) to extend effective usage. The cost of the system components is also worth noting: our prototype’s hardware (Jetson Nano board, camera module, GPS, etc.) costs only a few hundred dollars in total, which is relatively low compared to specialized devices, e.g., Envision or OrCam (which can cost a few thousand dollars). This suggests that with economies of scale, a commercial version of our solution could be made quite affordable for users or subsidizing organizations.

### Comparison with other systems

In terms of functionality, our all-in-one system covers a broader feature set than most existing solutions, which include object detection, obstacle distance estimation, scene captioning, text reading and GPS location sharing. By contrast, sensor-based canes usually offer only obstacle distance feedback; smartphone apps, e.g., Seeing AI offer object, text, and scene recognition but no direct obstacle avoidance alerts (and no built-in safety communications); wearable devices such as OrCam focus on text and face recognition; and services, e.g., Be My Eyes handle a wide range of tasks, but require human intervention and a network connection. Our system’s unique value is in integrating all these aspects as the AI provides immediate autonomous assistance and the IoT connectivity provides backup human assistance and orientation (GPS) services. This integration was reflected in user testing feedback. In performance comparisons, our system achieves state-of-the-art results on key metrics for edge devices. For example, our object detection accuracy and speed were on par with the best results reported by Pydala et al. (2024) for their Visisense IoT cane. They reported a 17 ms processing time per frame with  99% accuracy in controlled tests, using a combination of cloud and edge computing. Our system, running fully on edge (except when using the optional remote assistance), reached a slightly higher per-frame latency (in the tens of milliseconds) with an accuracy sufficient for practical use, which is impressive considering we did not rely on cloud offloading.

In energy consumption, Visisense had a very efficient profile (low energy per inference) by splitting workloads; our fully-edge approach uses more energy on the device, but this is the trade-off for being cloud-independent. This illustrates that edge AI has matured to the point where even complex tasks such as image captioning and language understanding can be done locally, albeit with careful model selection. In fact, we experimented with various Large Language Models (LLMs) on the device (as part of an AI voice assistant feature for querying the system). We benchmarked smaller LLMs that could run on the Jetson and found that certain optimized models could answer questions reasonably well, but larger models were too slow or resource-heavy. The performance results from Table 2 show that while edge devices can host LLMs, there is a significant speed/accuracy trade-off among models. This is a cutting-edge aspect of our solution; few assistive devices have a built-in conversational AI. The inclusion of an LLM-driven voice assistant in our system was exploratory, but it points toward future directions where users might have an interactive AI guide in addition to automated alerts.

### Practical implementation challenges

Building and testing the system revealed several challenges that need consideration for real-world deployment. One major challenge is the interface and usability. We learned that just because the system can provide a lot of information doesn’t mean it should do so all at once. If the device overloads the user with continuous speech (e.g., “car on left, tree ahead, person on right, sign overhead...” all in a stream), it can overload and confuse more than help. Finding the right balance of information is crucial. In our user trials, we tuned the verbosity via settings using an “exploration mode” which might describe the surroundings in more detail when the user is curious and queries for assistance. This kind of context-aware output is important to make the device a helpful aid rather than a distraction. Another challenge is the audio interface: using a speaker is fine in quiet areas, but in noisy city streets, the user may prefer bone-conduction headphones or earbuds to clearly hear the device. We must ensure that the device’s audio alerts do not prevent the user from hearing important environmental sounds (e.g., traffic or someone speaking to them). This remains an area for fine-tuning; for instance, using more directional audio cues (spatialized sound indicating where an obstacle is) could convey information in a less intrusive way than verbose descriptions.

Hardware-wise, there were challenges in form factor and weather proofing. Our prototype was a small package (Jetson dev kit, a battery pack and a camera mounted at chest level). This is acceptable as a prototype, but for daily use it should be lighter and more discreet; possibly integrated into a harness, cane, or eyewear. Devices such as Envision glasses are extremely compact and comfortable to wear but are less featured. There is a trade-off between power and size; our device is more powerful but slightly larger. We also have to consider heat: the Jetson can get warm if it has to work hard for an extended period. We added a heat-sink and mini fan, which fixed the overheating problem but added a bit of fan noise (not ideal). Later versions can utilize the Jetson in a reduced power mode or another board to reduce heat. Making the camera and electronics weatherproof (e.g., rain and dust) is also a primary requirement.

### Privacy and security

We designed the communication features with security in mind because privacy is a major concern for users in modern days. The idea of wearing a camera that is always analyzing the environment can raise questions, both for the user and for people around them, about how the data is used. By doing on-device processing, we ensure that raw video is not uploaded to any server for the AI functions. We adhered to guidelines to protect any personal data. For example, location data and live streams are sent to trusted contacts only who are connected to the private network with access that the user selects. In later versions, we might add face recognition for familiar individuals so the device could announce the person who’s approaching, but that opens other privacy concerns (maintaining a secure face database, preventing misuse). For now, we have limited our system to simple functionality that clearly has user consent. Overall, the analysis of our findings concludes that the suggested IoT-edge approach overcomes numerous drawbacks of previous technologies by offering deeper environmental insight than sensor-only devices, quicker and more personal responses compared to cloud-reliant applications, as well as a more unified user experience than single purpose alternatives. It clearly shows that an AI-driven, portable assistant for the blind is viable with the edge computing technologies of today. The comparisons with existing solutions show that no single solution addresses all needs; each has its strengths and weaknesses, but our solution offers a nice compromise by encompassing a number of features and support modes. We have also identified areas for improvement (user interface, form factor, additional sensors for edge cases). Ultimately, the positive feedback from evaluation users and the quantitative performance metrics give us confidence that this approach is a step forward in assistive technology, bringing us closer to a device that can function as a “digital guide dog” or co-pilot for blind and low-vision individuals in everyday life.

## Limitations and future work

The proposed system demonstrates promising real-time assistive performance; however, several limitations remain and define the next research priorities. In particular, improvements are needed in motion-aware robustness, hardware miniaturization, model capability under tight edge constraints, multimodal interaction design, and broader real-world validation. This section summarizes the main current limitations and outlines concrete future directions.

### Motion-aware navigation

Although the current work provides strong perception and interaction results, motion-aware navigation robustness is still limited. Specifically, we do not yet report a dedicated quantitative analysis of heading error, drift accumulation over walking distance, or sensitivity to device-pose variability. This should be considered when interpreting navigation reliability in dynamic trajectories. As a next step, we will benchmark motion robustness using yaw mean absolute error, drift per traveled distance, and path-deviation metrics under controlled pose perturbations. We will also evaluate lightweight IMU-vision fusion baselines on the same edge platform to improve robustness while preserving latency and power constraints.

### Hardware miniaturization and efficiency

One immediate goal is to make the system lighter, smaller, and more power-efficient. The current prototype, though portable, can be refined into a more wearable form factor. Future versions might use smaller PCB integrations or system-on-module designs to reduce size as well as exploring alternative edge AI hardware, which is also worthwhile. Special low-power AI chips (NPUs) that are only just beginning to appear in smartphones and AR glasses can be used to move from hours of battery life to all-day operation. Additionally, battery technology and power management can be tweaked, e.g., dynamic power scaling (where the phone can downclock or disable some parts during the time when the user is idle or environments are static) in order to save power. Advances in thermal management (passive cooling designs) will also play a role in removing the need for fans, thereby reducing noise and power draw. The final aim is to integrate the core components into a device embedding a pair of intelligent glasses or a small unit that can be clipped onto a cane or belt, making it effectively invisible and unobtrusive to the user.

Enhanced and New AI Models: On the software side, continual advancements in AI will allow us to expand and improve the device’s functionalities. One area is object detection and recognition. We plan to train and incorporate more classes of objects, including specific categories relevant to blind users (e.g., detecting crosswalk indicators, road signs, elevator buttons, entrance doors versus windows, etc.). Improving the detection of non-rigid obstacles, e.g., overhanging branches or half-open doors, could be achieved with advanced vision transformers or hybrid models that consider temporal information from video. Another promising avenue is image segmentation; instead of just bounding boxes, the device could segment the walkable area vs. the obstacle area in the view and convey that to the user (possibly through augmented audio or haptic feedback). For scene understanding, larger visual-language models (e.g., the latest image captioning networks or multimodal transformers) could provide more detailed and human-like descriptions. As edge hardware improves, newer and bigger models might eventually run locally. Similarly, the conversational AI (LLM) component can be improved. In our prototype we used a relatively small model for the voice assistant. In the future, we could use larger models or even mulimodal LLM, effectively making the device a digital assistant as well as a navigator^42^.

Another future AI feature is facial recognition of known people (friends, family) to alert the user when someone familiar is nearby. Reading emotional cues or body language (is anyone waving at me? is anyone sad or pleased?) could introduce a social factor into the assistance, though that’s advanced-level. Also, text reading can be expanded: aside from OCR, we could have the system read signs and symbols (e.g., logos, directional arrows, etc.) and read them back in kind (“the sign indicates an exit to your left”). All these developments in AI are towards making the device more context-sensitive and useful in more contexts.

### Multimodal sensing and integration

On top of that, the use of other sensing modalities beyond the Intel RealSense D455 is also a possibility. While the D455 has the capability of stereo depth sensing up to 6 meters, the use of a higher-range stereo camera, e.g., the Stereolabs ZED 2, which is capable of detecting up to 20 meters, would significantly be able to provide long-distance obstacle detection^43^. This extended depth perception is particularly valuable for identifying crosswalks, poles, or approaching vehicles at a distance, allowing for earlier warnings and safer navigation. Additionally, A major improvement in scene captioning can be achieved by combining a stereo camera with Vision-Language Models (VLMs). For example, if the stereo camera detects an object, the VLM can classify it as a “traffic sign,” “stationary barrier,” or “approaching cyclist”, refining navigation instructions for visually impaired users. An additional critical enhancement is fall detection using an Inertial Measurement Unit (IMU). With accelerometer and gyroscope data analysis, the system is able to detect sudden orientation change, braking, or abnormal motion patterns, which could signify a fall^44^. The feature can be integrated to initiate an automatic alert to emergency services or caregivers or give immediate audio feedback to the user, requesting him or her to confirm if assistance is needed, as well as log fall events for additional research to maximize user safety. For external navigation, integration of GPS and IMU information with mapping capabilities would enhance real-time turn-by-turn directions. GPS can be used by the system to determine user position, while the vision system confirms the surroundings for accuracy, e.g., ensuring the user is at an allotted crosswalk prior to proceeding.

### Improved user interaction and HCI

As we continue to build the device, a key focus is enhancing human-computer interaction. Instead of relying solely on speech output, we want to add stereo sound-based 3D localization with spatialized audio^45^. That will allow users to intuitively perceive obstacles; objects that are closer will be louder, with direction conveyed through binaural audio (e.g., a bicycle approaching from the right will be in the right ear). This affords continuous awareness with no requirement for ongoing verbal prompts. To further support responsiveness, haptic feedback will provide silent real-time notification in the form of a vibration belt, vest, or wearable sensors. The system will trigger vibrations on the same side (left-side vibration for an object to the left), resulting in a multimodal assistive system that is able to increase user confidence and safety.

We also wish to customize the user experience. Users vary in their needs and wants; some will desire complete running commentary, others minimal notification. Upcoming software will enable greater control over verbosity, voice quality, languages, and what types of objects to speak about. AI can even be taught based on the user’s behavior; e.g., if the user always ignores “storefronts” announcements but always responds to “traffic approaching”, the system can prioritize the latter. Another future feature is logging and analytics: the device can keep a personal record of obstacles overcome, places visited, etc., to aid in rehabilitation or training. For example, mobility trainers could review these logs to determine where a user is having the most trouble and adjust training accordingly. This, of course, would be done with the user’s permission and with privacy controls (data could be stored locally or encrypted).

### Integration with smart cities and IoT infrastructure

Looking beyond the individual device, there is great potential in integrating assistive devices with the broader smart city infrastructure. In the future, our device could communicate with traffic signals to know when the pedestrian light is green or receive data from connected vehicles, such as a public bus or tram, otherwise silent. It is possible to detect dedicated signage and seamlessly incorporate information to enable access to real-time transit data. Another concept is leveraging edge computing in the environment (sometimes called Mobile Edge or Fog computing). While our device is self-contained, there may be scenarios where offloading to a nearby edge server (e.g., a 5G base station with computing) could provide a boost for heavy tasks. For example, a wearable might normally do everything on-board, but if it’s in range of a trusted edge compute hotspot (say, provided at a public venue), it could temporarily use that to run a very large AI model for a harder task (e.g., detailed scene reconstruction). This kind of dynamic IoT ecosystem could keep the wearable light while giving access to more power when available. Research on distributed computing for assistive devices (e.g., a cane that talks to a home server) has shown improvements in performance without sacrificing latency too much^46^.

### User studies and accessibility research

Future work will also involve rigorous field testing and user studies assisted by the blind community. We plan to do longitudinal studies wherein users can bring the device back to their home and integrate it into their daily routine for several weeks and then provide feedback to us as to what they find most valuable and what doesn’t work. This field feedback is invaluable to detect usability flaws that short laboratory tests might not detect. We are also excited to learn more about how users typically interact with the device, i.e., are they constantly using it or just when they transition to another area, or how quickly do they learn to trust its notifications, or under what circumstances do they become confused or wrong? From that input, we can further develop both the hardware (ergonomics, wearability) and software (UI, feature set)^47^.

## Conclusion

This research successfully creates an edge computing and IoT-based real-time vision assistance system to improve mobility and independence for the visually impaired. Unlike traditional sensor-based systems, our solution integrates deep learning for scene awareness, real-time object detection, and remote assistance with low latency and privacy. Running processing on the device using Jetson Orin Nano does away with our reliance on the cloud. This makes the solution faster, more secure, and cheaper. Our system has proven very accurate according to the tests, and it uses power efficiently while being compatible with a great number of assistive features. Future enhancements include streamlining the user interface for better feedback, optimizing AI models to achieve faster real-time performance, and intermingling different types of data (e.g., LiDAR and audio) for improved environmental perception. The proposed IoT-Edge Vision Assistant represents a major advancement toward intelligent and autonomous vision assistance, offering a useful, versatile, and functional application to the visually impaired.

## Data Availability

All data generated or analyzed during this study will be made available on request. The source codes are publicly available at: https://github.com/akashshingha850/SENSEYE-Resource-Aware-Visionary-Framework.

## References

[1] Gbenga, D. E., Shani, A. I. & Adekunle, A. L. Smart walking stick for visually impaired people using ultrasonic sensors and arduino. *Int. J. Eng. Technol.* **9**, 3435–3447 (2017).

[2] Elmannai, W. & Elleithy, K. Sensor-based assistive devices for visually-impaired people: Current status, challenges, and future directions. *Sensors* **17**, 565 (2017). 28287451 10.3390/s17030565 PMC5375851

[3] Payne, J. Microsoft launches ai app for visually impaired. UWIRE Text 1–1 (2017).

[4] Nguyen, X.-T.-A., Koopman, J., van Genderen, M. M., Stam, H. L. & Boon, C. J. Artificial vision: The effectiveness of the orcam in patients with advanced inherited retinal dystrophies. *Acta Ophthalmol.* **100**, e986–e993 (2022). 34569160 10.1111/aos.15001 PMC9292690

[5] Rahman, M. A. & Sadi, M. S. Iot enabled automated object recognition for the visually impaired. *Comput. Methods Programs Biomed. Update* **1**, 100015 (2021).

[6] Wang, J., Wang, S. & Zhang, Y. Artificial intelligence for visually impaired. *Displays* **77**, 102391 (2023).

[7] Ulfa, A., Rosspertiwi, A. A., Sahroni, A. & Murnani, S. S-cane: Ultrasonic sensor-based smart cane for the visually impaired. In *2023 IEEE International Biomedical Instrumentation and Technology Conference (IBITeC)* 7–11 (IEEE, 2023).

[8] Dambhare, S. & Sakhare, A. Smart stick for blind: Obstacle detection, artificial vision and real-time assistance via gps. In *2nd National Conference on Information and Communication Technology (NCICT)* vol. 2, 31–33 (2011).

[9] Oladayo, O. O. A multidimensional walking aid for visually impaired using ultrasonic sensors network with voice guidance. *Int. J. Intell. Syst. Appl.* **6**, 53–59 (2014).

[10] Wahab, M. H. A. et al. Smart cane: Assistive cane for visually-impaired people. arXiv preprint arXiv:1110.5156 (2011).

[11] Saaid, M. F., Ismail, I. & Noor, M. Z. H. Radio frequency identification walking stick (rfiws): A device for the blind. In *2009 5th International Colloquium on Signal Processing & Its Applications* 250–253 (IEEE, 2009).

[12] Joseph, A. M., Kian, A. & Begg, R. State-of-the-art review on wearable obstacle detection systems developed for assistive technologies and footwear. *Sensors* **23**, 2802 (2023). 36905003 10.3390/s23052802 PMC10007677

[13] Singh, D. K. Dynamic environment exploration tool: A blind’s eye. In *Proceedings of the XYZ Conference* (2015).

[14] Granquist, C. et al. Evaluation and comparison of artificial intelligence vision aids: Orcam myeye 1 and seeing ai. *J. Visual Impairment Blindness* **115**, 277–285 (2021).

[15] Tapu, R., Mocanu, B. & Zaharia, T. Deep-see: Joint object detection, tracking and recognition with application to visually impaired navigational assistance. *Sensors* **17**, 2473 (2017). 29143795 10.3390/s17112473 PMC5713031

[16] Murali, V. N. & Coughlan, J. M. Smartphone-based crosswalk detection and localization for visually impaired pedestrians. In *2013 IEEE International Conference on Multimedia and Expo Workshops (ICMEW)* 1–7 (IEEE, 2013). 10.1109/ICMEW.2013.6618432 PMC4210954 25360440

[17] Pydala, B., Kumar, T. P. & Baseer, K. K. Visisense: a comprehensive iot-based assistive technology system for enhanced navigation support for the visually impaired. *Scalable Comput. Pract. Exp.* **25**, 1134–1151 (2024).

[18] Saputra, M. R. U., Santosa, P. I. et al. Obstacle avoidance for visually impaired using auto-adaptive thresholding on kinect’s depth image. In *2014 IEEE 11th Intl Conf on Ubiquitous Intelligence and Computing and 2014 IEEE 11th Intl Conf on Autonomic and Trusted Computing and 2014 IEEE 14th Intl Conf on Scalable Computing and Communications and Its Associated Workshops* 337–342 (IEEE, 2014).

[19] Sethuraman, S. C., Tadkapally, G. R., Mohanty, S. P., Galada, G. & Subramanian, A. Magiceye: An intelligent wearable towards independent living of visually impaired. arXiv preprint arXiv:2303.13863 (2023).

[20] Ye, J., Mansour, A. & Huang, F. Enhancing real-time heading estimation for pedestrian navigation via deep learning and smartphone embedded sensors. *Sci. Rep.* **15**, 31672 (2025). 40866377 10.1038/s41598-025-13390-9 PMC12391363

[21] Waisberg, E. et al. Meta smart glasses-large language models and the future for assistive glasses for individuals with vision impairments. *Eye* **38**, 1036–1038 (2024). 38049627 10.1038/s41433-023-02842-z PMC11009354

[22] Avila, M., Wolf, K., Brock, A. & Henze, N. Remote assistance for blind users in daily life: A survey about be my eyes. In *Proceedings of the 9th ACM International Conference on PErvasive Technologies Related to Assistive Environments* 1–2 (2016).

[23] Shi, W., Cao, J., Zhang, Q., Li, Y. & Xu, L. Edge computing: Vision and challenges. *IEEE Internet Things J.* **3**, 637–646 (2016).

[24] Archet, A., Gac, N., Orieux, F. & Ventroux, N. Embedded ai performances of nvidia’s jetson orin soc series. In *17ème Colloque National du GDR SOC2* (2023).

[25] Gamess, E. & Hernandez, S. Performance evaluation of different raspberry pi models for a broad spectrum of interests. *Int. J. Adv. Comput. Sci. Appl.* **13** (2022).

[26] Radford, A. et al. Robust speech recognition via large-scale weak supervision. In *International Conference on Machine Learning* 28492–28518 (PMLR, 2023).

[27] Lewis, P. et al. Retrieval-augmented generation for knowledge-intensive nlp tasks. *Adv. Neural. Inf. Process. Syst.* **33**, 9459–9474 (2020).

[28] Douze, M. et al. The faiss library. *IEEE Trans. Big Data* (2025).

[29] Yang, A. et al. Qwen3 technical report. arXiv preprint arXiv:2505.09388 (2025).

[30] Hübner, P., Hou, J. & Iwaszczuk, D. Evaluation of intel realsense d455 camera depth estimation for indoor slam applications. *Int. Arch. Photogramm. Remote. Sens. Spat. Inf. Sci.* **48**, 1207–1214 (2023).

[31] Sandler, M., Howard, A., Zhu, M., Zhmoginov, A. & Chen, L.-C. Mobilenetv2: Inverted residuals and linear bottlenecks. In *Proceedings of the IEEE Conference on Computer Vision and Pattern Recognition* 4510–4520 (2018).

[32] Lin, T.-Y. et al. Microsoft coco: Common objects in context. In *European Conference on Computer Vision* 740–755 (Springer, 2014).

[33] Liu, W. et al. Ssd: Single shot multibox detector. In *European Conference on Computer Vision* 21–37 (Springer, 2016).

[34] Jeong, E., Kim, J. & Ha, S. Tensorrt-based framework and optimization methodology for deep learning inference on jetson boards. *ACM Trans. Embedded Comput. Syst. (TECS)* **21**, 1–26 (2022).

[35] Bordes, F. et al. An introduction to vision-language modeling. arXiv preprint arXiv:2405.17247 (2024).

[36] Liu, Z. et al. Nvila: Efficient frontier visual language models. In *Proceedings of the Computer Vision and Pattern Recognition Conference* 4122–4134 (2025).

[37] Quincozes, S., Emilio, T. & Kazienko, J. Mqtt protocol: Fundamentals, tools and future directions. *IEEE Lat. Am. Trans.* **17**, 1439–1448 (2019).

[38] Grinberg, M. *Flask Web Development* (O’Reilly Media, Inc., 2018).

[39] Crickard III, P. *Leaflet js essentials* (Packt Publishing Ltd, 2014).

[40] Sredojev, B., Samardzija, D. & Posarac, D. Webrtc technology overview and signaling solution design and implementation. In *2015 38th International Convention on Information and Communication Technology, Electronics and Microelectronics (MIPRO)* 1006–1009 (IEEE, 2015).

[41] Aboyomi, D. D. & Daniel, C. A comparative analysis of modern object detection algorithms: Yolo vs. ssd vs. faster r-cnn. *ITEJ Inf. Technol. Eng. J.* **8**, 96–106 (2023).

[42] Hinck, M., Olson, M. L., Cobbley, D., Tseng, S.-Y. & Lal, V. Llava-gemma: Accelerating multimodal foundation models with a compact language model. arXiv preprint arXiv:2404.01331 (2024).

[43] Abdelsalam, A., Mansour, M., Porras, J. & Happonen, A. Depth accuracy analysis of the zed 2i stereo camera in an indoor environment. *Robot. Auton. Syst.* **179**, 104753 (2024).

[44] Zhang, C. C., Wang, C., Dai, X. & Liu, S. Camera-based analysis of human pose for fall detection. In *2023 Congress in Computer Science, Computer Engineering, & Applied Computing (CSCE)* 1779–1782 (IEEE, 2023).

[45] Wu, W.-C., Hsieh, C.-H., Huang, H.-C., Chen, O. T.-C. & Fang, Y.-J. Hearing aid system with 3d sound localization. In *TENCON 2007-2007 IEEE Region 10 Conference* 1–4 (IEEE, 2007).

[46] Júnior, M. J., Maia, O. B., Oliveira, H., Souto, E. & Barreto, R. Assistive technology through internet of things and edge computing. In *2019 IEEE 9th International Conference on Consumer Electronics (ICCE-Berlin)* 330–332 (IEEE, 2019).

[47] McDonnall, M. C., Steverson, A. & Boydstun, J. Actual and preferred methods for learning to use assistive technology. *Assist. Technol. Outcomes Benefits* **2024**, 20 (2024). 39290852 PMC11404533

